# Neonatal Hyperinsulinemia and Maternal Diabetes Associated With a Hepatocyte Nuclear Factor 4 Alpha (HNF4A) Variant

**DOI:** 10.7759/cureus.107058

**Published:** 2026-04-14

**Authors:** Yuki Takasugi, Ryuichi Nakagawa, Hideko Ono, Yasushi Nakajima, Kei Takasawa

**Affiliations:** 1 Department of Pediatrics, Japanese Red Cross Musashino Hospital, Tokyo, JPN; 2 Department of Pediatrics and Developmental Biology, Institute of Science Tokyo, Tokyo, JPN; 3 Department of Neonatology, Japanese Red Cross Musashino Hospital, Tokyo, JPN; 4 Department of Diabetes and Endocrinology, Nakajima Internal Medicine Clinic, Tokyo, JPN

**Keywords:** congenital hyperinsulinism, diabetes mellitus, genetic testing, hepatocyte nuclear factor 4 alpha, maturity-onset diabetes of the young

## Abstract

Maturity-onset diabetes of the young type 1 (MODY1) is caused by autosomal dominant variants in the hepatocyte nuclear factor 4 alpha (*HNF4A*) gene. This genetic defect results in a characteristic biphasic phenotype, typically manifesting as neonatal hyperinsulinism (HI) followed by the development of diabetes mellitus (DM) in young adulthood. HNF4A acts as a master transcriptional regulator for insulin gene expression and hepatic metabolic pathways, providing a molecular basis for understanding the link between neonatal hyperinsulinemic hypoglycemia and DM in young adulthood. Therefore, accurate molecular diagnosis is essential for appropriate clinical management. We report a case of a male infant who presented with neonatal HI and a family history of young-onset DM in his mother. Following the initial identification of a variant via next-generation sequencing, Sanger sequencing was utilized as the gold standard for familial segregation analysis to confirm the findings. The testing identified a novel heterozygous *HNF4A* variant, c.598G>C (p.Ala222Pro), in the proband and his mother. The variant was classified as likely pathogenic. The heterozygous state, characterized by the presence of one pathogenic and one wild-type allele, is consistent with an autosomal dominant inheritance pattern. This specific variant serves as the molecular fingerprint for the family’s MODY1 diagnosis. Identification of this variant enabled reconsideration of the mother's diabetes management, including potential therapeutic de-escalation from insulin. This case highlights that neonatal HI may facilitate the diagnosis of parental MODY1. Genetic testing for *HNF4A* variants should be considered in such cases, as it may broaden therapeutic options and support individualized management of DM.

## Introduction

Maturity-onset diabetes of the young (MODY) is an inherited form of non-autoimmune diabetes mellitus (DM) that typically presents during adolescence or young adulthood [1]. It is estimated to account for 1% of all cases of diabetes, making accurate molecular diagnosis essential for optimal patient management [2]. MODY is transmitted in an autosomal dominant manner, resulting in strong inheritance patterns across generations [1]. It means that a heterozygous variant (a mutation in only one of the two copies of a gene) is sufficient to cause the condition. The clinical presentation and treatment response vary considerably among the different genetic subtypes of MODY, underscoring the importance of a correct molecular diagnosis.

Among the most common forms of monogenic diabetes are those caused by variants in the transcription factors hepatocyte nuclear factor 1 alpha (HNF1A; MODY3) and hepatocyte nuclear factor 4 alpha (HNF4A; MODY1). MODY1 is caused by variants in the *HNF4A* gene, which encodes a nuclear transcription factor that plays a critical role in the development and function of pancreatic beta cells and the liver [1]. Its expression is controlled by two distinct promoters: P1 (liver-specific) and P2 (pancreas-specific) [1]. In pancreatic beta cells, the P2-driven isoforms (HNF4A7-9) are predominant and are essential for maintaining beta-cell identity and glucose-stimulated insulin secretion [3]. The temporal and tissue-specific usage of these promoters provides a molecular basis for the complex clinical presentation of *HNF4A* variants. Variants in this gene typically lead to progressive beta-cell dysfunction and subsequent hyperglycemia [1].

Importantly, *HNF4A* variants are characterized by a "biphasic phenotype," a clinical course consisting of two distinct phases: an initial period of insulin oversecretion in the neonate, followed by insulin-deficient diabetes later in life. From a diagnostic perspective, clinicians should recognize specific “red flags” that raise clinical suspicion of *HNF4A*-related disorders. These include significantly increased birth weight (macrosomia) and a strong multi-generational family history of early-onset diabetes. Recognizing these features is critical because patients with MODY1 are highly sensitive to low-dose sulfonylurea [4], and the diagnosis allows consideration of alternative therapeutic options to insulin. Clarifying the full clinical spectrum, from transient neonatal hyperinsulinism (HI) to adult-onset diabetes, is essential for improving clinical pathways and personalized treatment strategies. Here, we report a case in which maturity-onset diabetes of the young 1 (MODY1) was diagnosed in the mother following the detection of HI in her child, highlighting the importance of genetic analysis in families suspected of having *HNF4A* variants.

## Case presentation

Regarding family history, the mother of the proband was 27 years old at the time of delivery. She had been diagnosed with diabetes at 17 years of age (well before 25 years of age), a chronological feature highly characteristic of MODY1. Her DM was non-obese (hemoglobin A1c, 72.69 mmol/mol (International Federation of Clinical Chemistry and Laboratory Medicine (IFCC)). At the time of diagnosis, laboratory findings, including a fasting blood C-peptide immunoreactivity (CPR) level of 0.36 nmol/L, and a CPR index (fasting blood CPR×100/fasting blood glucose) of 0.74, suggested a mild decline in insulin secretion, whereas anti-glutamic acid decarboxylase antibodies were negative. Given the early onset of diabetes and potential future pregnancy, intensive insulin therapy with multiple daily injections was initiated as first-line treatment. The maternal grandfather also had DM with onset during adolescence (further details are unknown).

**Figure 1:**
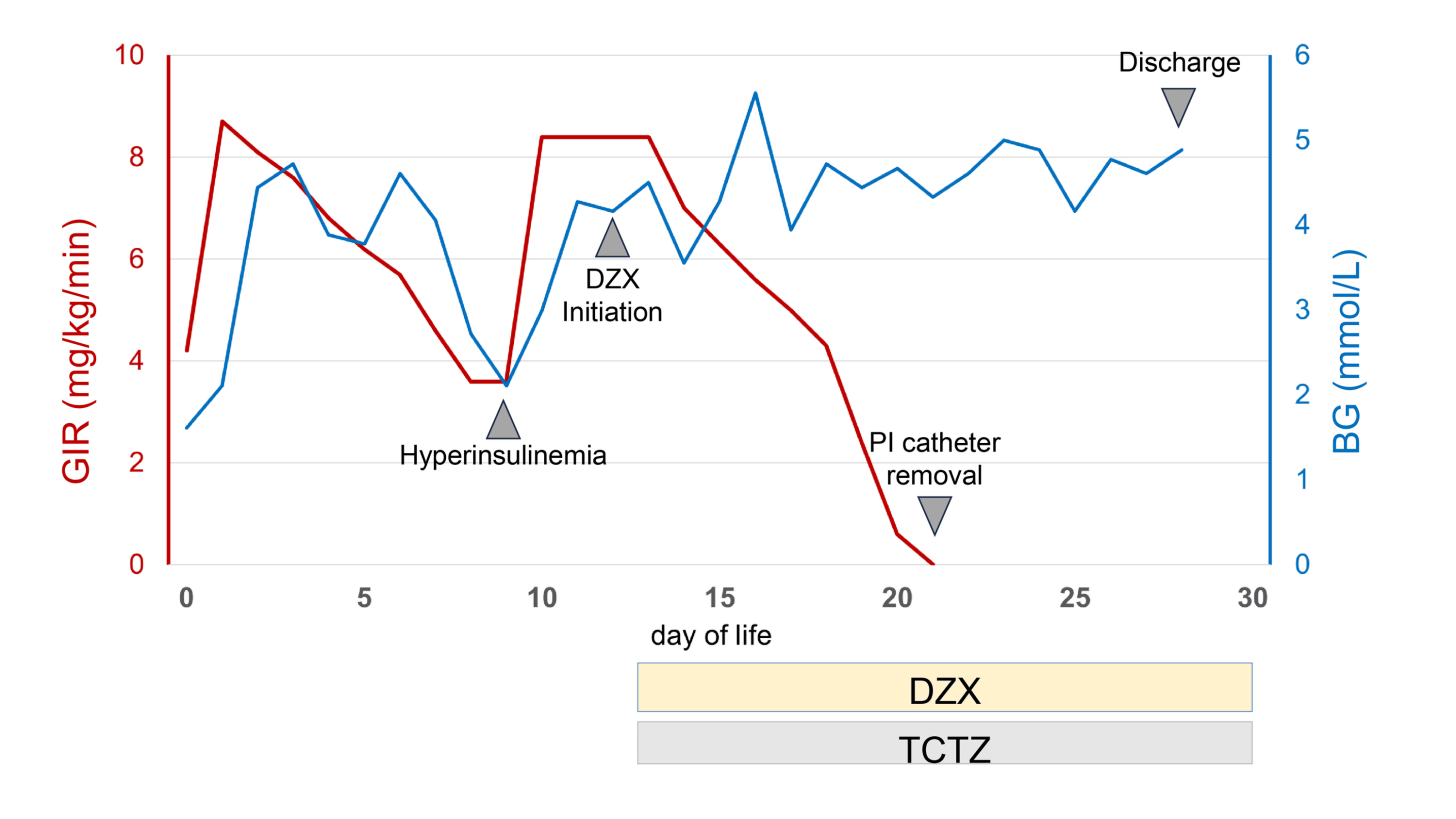
Clinical course of the proband from birth to discharge, demonstrating neonatal HI. The graph illustrates the management of BG levels (blue line) and the required GIR (red line) over the first 30 days of life. Initially, the patient exhibited hypoglycemia requiring a high GIR (maximum 8.7 mg/kg/min) to maintain normoglycemia. On day nine of life, a diagnosis of HI was confirmed. Following the initiation of DZX (5 mg/kg/day) and TCTZ on day 12 of life, BG stabilized, enabling an increase in oral milk intake and a gradual reduction of the GIR, which was discontinued by day 21 of life. The patient was discharged on day 28 of life on oral DZX therapy. The horizontal bars at the bottom of the figure indicate the respective periods of administration for DZX and TCTZ until discharge. BG, blood glucose; GIR, glucose infusion rate; HI, hyperinsulinism; DZX, diazoxide; TCTZ, trichloromethiazide; PI, peripheral intravenous.

Born to this mother with a clinical phenotype strongly suggestive of monogenic diabetes, the proband, a male infant, was delivered at 39 weeks and 6 days of gestation with a birth weight of 3,520 g (+1.5 standard deviations, large for gestational age) and a birth height of 47 cm (-1.2 standard deviations). The patient was admitted to the neonatal intensive care unit for management of hypoglycemia (Table 1). No internal or external malformations were identified, including abnormalities of the abdominal organs or kidneys. Furthermore, no systemic complications or biochemical abnormalities, including hypocholesterolemia or renal tubulopathy, were observed. Persistent hypoglycemia, with blood glucose levels in the 1.11 mmol/L range upon admission, prompted initiation of a high-concentration glucose infusion via a peripheral intravenous catheter, reaching a maximum glucose infusion rate (GIR) of 8.7 mg/kg/min (Figure 1). As oral feeding volume increased and the GIR was subsequently tapered, there was recurrent hypoglycemia (2.11 mmol/L) on day nine of life, necessitating a further increase in the GIR. Laboratory test results obtained later revealed an immunoreactive insulin (IRI) level of 83.34 pmol/L during the hypoglycemic event on day nine of life, confirming the diagnosis of HI. Diazoxide was initiated at a dose of 18 mg (5 mg/kg/day) on day 12 of life. Following initiation of diazoxide, glycemic control improved markedly. The patient maintained euglycemia despite the increase in oral milk feeding volume and the concurrent reduction in intravenous GIR. The peripheral intravenous catheter was removed on day 21 of life, and the patient was discharged home on day 28 of life. The patient continued receiving a stable dose of diazoxide until 18 months of age, resulting in a relative dose reduction as body weight increased. During this period, no significant hypoglycemic episodes were observed, and physical growth and psychomotor development remained normal. Key laboratory results of the proband and the mother are shown in Table 2.

**Table 1:** Clinical parameters and timing of neonatal events in the proband. The table summarizes the essential anthropometric data and the precise timeline of clinical presentation following delivery. The proband's birth weight was large for date (+1.5 SD), a clinical hallmark of *HNF4A*-related neonatal HI. Notably, the onset of hypoglycemia occurred within the first few hours of life (2 hours and 6 minutes post-delivery), highlighting the time-sensitive nature of hyperinsulinemic symptoms in this case. HNF4A, hepatocyte nuclear factor 4 alpha; SD, standard deviation; HI, hyperinsulinism; NA, not applicable.

Parameter	Value	Z-score
Gestational age	39 weeks and 6 days	NA
Time of birth	14:13	NA
Birth weight	3,520 g	+1.5 SD
Birth height	47 cm	-1.2 SD
Onset of hypoglycemia	16:19 (2h 6min after birth)	NA

**Table 2:** Laboratory findings of the proband and the mother. The table presents the critical laboratory findings for both the proband and the mother. In the proband, HI was confirmed on day nine of life by the presence of inappropriately elevated IRI (83.34 pmol/L). For the mother, an elevated HbA1c level (72.69 mmol/mol) indicates chronic hyperglycemia. Furthermore, the CPR index of 0.74 suggests a relative deficiency in insulin secretion, which is a characteristic of *HNF4A*-related diabetes. BG, blood glucose; GIR, glucose infusion rate; IRI, immunoreactive insulin; HbA1c, hemoglobin A1c; CPR, C-peptide immunoreactivity; CPR index, fasting blood CPR×100/fasting blood glucose; HI, hyperinsulinism; HNF4A: hepatocyte nuclear factor 4 alpha.

Parameter		Result	Reference range
The proband	BG upon admission (mmol/L)	1.11	>2.76
	BG after GIR tapered (mmol/L)	2.11	>2.76
	IRI with hypoglycemia on day nine of life (pmol/L)	83.34	<12
The mother	HbA1c (mmol/mol)	72.69	<47.5
	CPR (nmol/L)	0.36	0.17-0.66
	CPR index	0.74	>0.8

**Figure 2:**
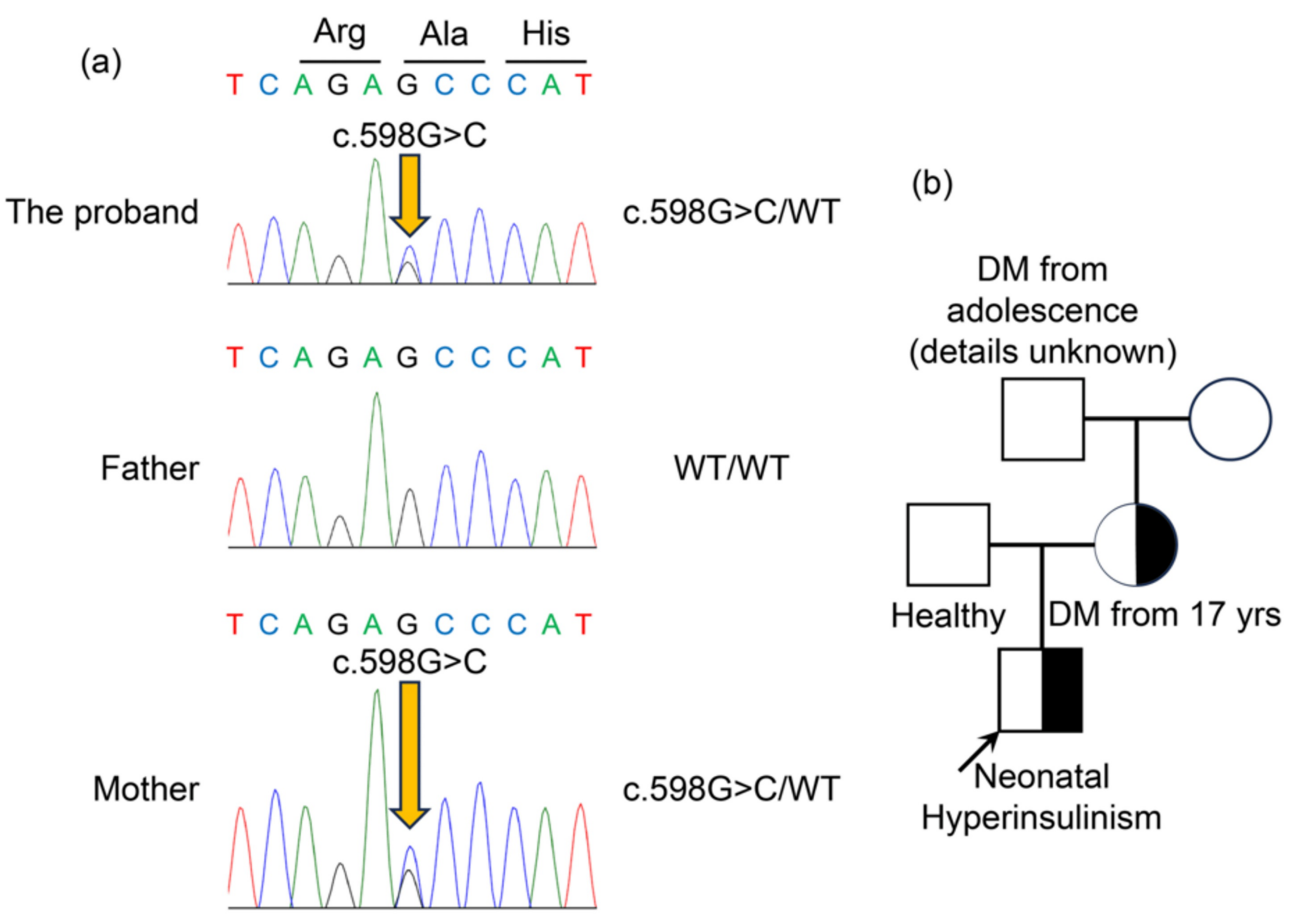
Molecular genetic analysis and three-generation pedigree of the affected family. (a) Sanger sequencing chromatograms identifying a novel heterozygous *HNF4A* variant. The electropherograms display the nucleotide sequence at the site of the c.598G>C variant, which results in the p.Ala222Pro amino acid substitution. Both the proband and the mother exhibit a characteristic heterozygous double peak (G and C) at the specific locus, whereas the father shows a homozygous WT sequence (single G peak). (b) The family pedigree demonstrates an autosomal dominant inheritance pattern. The proband (indicated by the arrow) presented with neonatal HI. The mother, carrying the same heterozygous variant (c.598G>C/WT), was diagnosed with DM at age 17. The maternal grandfather also reported early-onset diabetes from adolescence (clinical details unknown), further supporting the autosomal dominant inheritance and the diagnosis of MODY1 within the family. Symbols: Squares represent males; circles represent females; and filled/half-filled symbols indicate affected family members carrying the *HNF4A* variant. HNF4A, hepatocyte nuclear factor 4 alpha; WT, wild-type; HI, hyperinsulinism; DM, diabetes mellitus; MODY1, maturity-onset diabetes of the young type 1; Arg, arginine; Ala, alanine; His, histidine.

**Figure 3:**
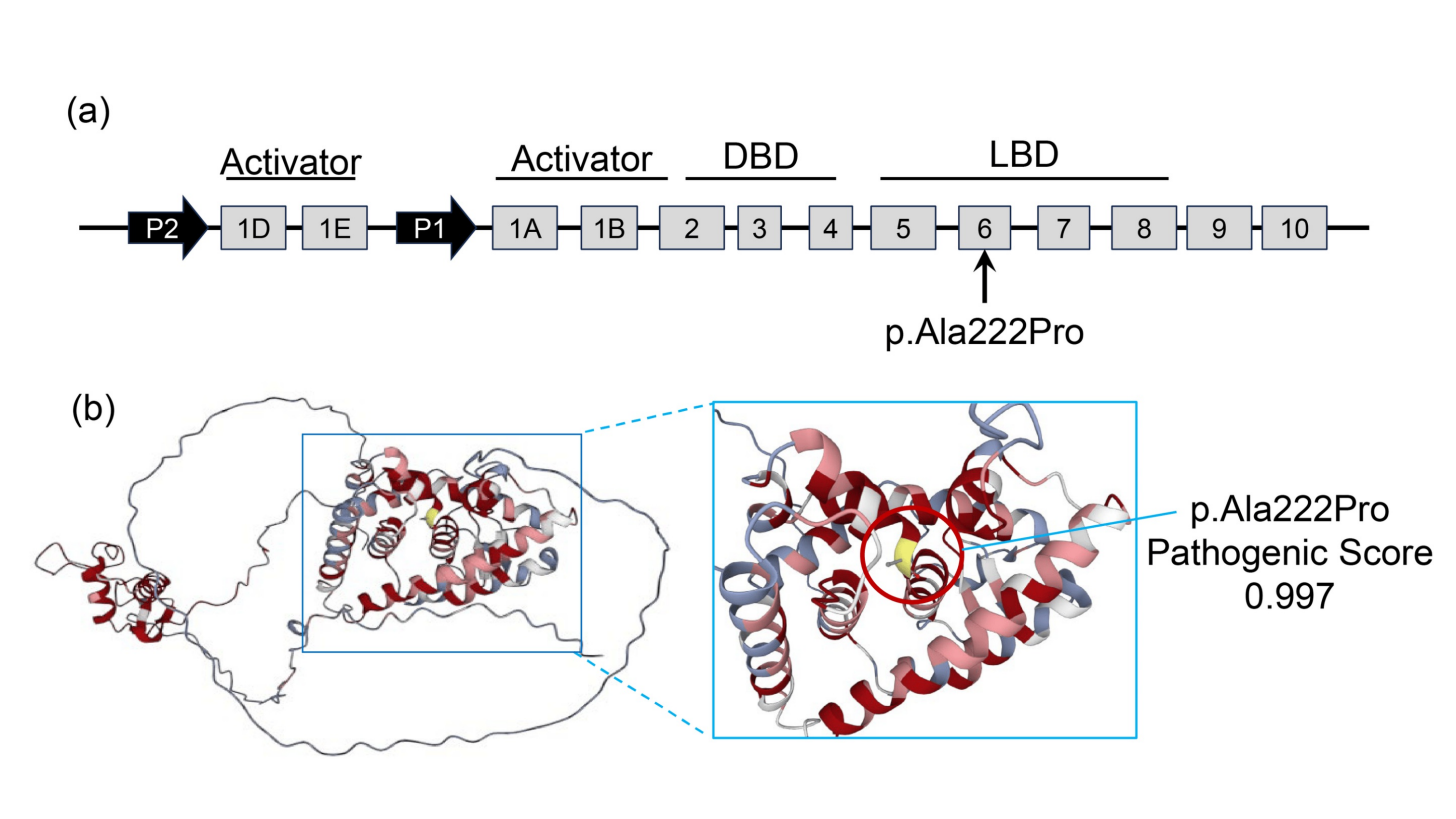
Structural localization and computational pathogenicity prediction of the novel HNF4A p.Ala222Pro variant. (a) Genomic structure of *HNF4A* and location of the p.Ala222Pro variant. The *HNF4A* gene expression is controlled by two alternative promoters: P1 (predominant in the liver) and P2 (predominant in pancreatic beta cells). The identified p.Ala222Pro variant is located within exon 6, which encodes a critical portion of the LBD. The LBD is essential for the transcriptional activity and protein dimerization of HNF4A. Numbers in gray boxes represent exons, while functional regions include the DBD and activation domains. Image credit: The authors, using Microsoft PowerPoint (Microsoft Corporation, Redmond, WA, US). (b) Three-dimensional protein modeling and pathogenicity assessment by AlphaMissense [6]. The variant is mapped onto the HNF4A* *protein structure. AlphaMissense analysis assigned a pathogenic score of 0.997 to the p.Ala222Pro substitution. This score, being near the maximum value of 1.0, strongly indicates that the mutation is "likely pathogenic." The red-colored regions in the 3D model represent areas with high pathogenicity scores, highlighting that the variant occurs in a highly conserved and functionally sensitive site. Image Credit: Created by the authors using AlphaMissense under the Creative Commons Attribution-NonCommercial-ShareAlike 4.0 International (CC BY-NC-SA 4.0) license [6]. *HNF4A*, hepatocyte nuclear factor 4 alpha; Ala, alanine; Pro, proline; LBD, ligand-binding domain; DBD, DNA-binding domain.

Based on the strong family history of young-onset DM and the presence of HI in the infant, MODY1 or MODY3 was suspected. Genetic testing of the proband was performed at the Kazusa DNA Research Institute using next-generation sequencing. Following identification of a specific variant of the proband, Sanger sequencing of family members was conducted. Genetic analysis revealed a heterozygous c.598G>C variant (p.Ala222Pro) of the *HNF4A* gene in both the patient and the mother (Figure 2). The identified variant is located in exon 6 of the *HNF4A* gene, which corresponds to the ligand-binding domain (Figure 3a). This variant had not been previously registered in established databases, such as the Human Gene Mutation Database (HGMD) or the Clinical Variation Database (ClinVar). Pathogenicity was predicted using multiple in silico prediction tools, including PolyPhen-2, AlphaMissense, and the Combined Annotation Dependent Depletion (CADD) score [5-7]. PolyPhen-2 classified it as probably damaging (score, 0.978; sensitivity, 0.76; specificity, 0.96), AlphaMissense predicted it to be likely pathogenic (score, 0.997) (Figure 3b), and the CADD score was significantly high (raw score, 5.595578; PHRED, 32). According to the American College of Medical Genetics and Genomics guidelines (ACMG) [8], the variant was classified as likely pathogenic. This variant was therefore considered the cause of HI in the patient and the young-onset diabetes in the mother. The mother is currently undergoing gradual reduction of insulin dosage while receiving concomitant therapy with a dipeptidyl peptidase-4 (DPP-4) inhibitor.

## Discussion

In the present case, the diagnosis of MODY1 in the mother was prompted by the detection of HI in her child. Similar parent-child presentations, in which neonatal manifestations lead to the diagnosis of diabetes in a parent, have been increasingly reported in the literature [9,10]. In clinical reviews of MODY, it is rare for parents of infants with HI to have a pre-existing diagnosis of MODY1; rather, they are frequently misdiagnosed with type 2 diabetes or gestational diabetes [4]. Therefore, the diagnosis of HI in a neonate should prompt a thorough evaluation of parental glycemic status or a re-evaluation of the specific diabetes subtype.

Heterozygous variants in the *HNF4A* gene are known to cause a characteristic biphasic phenotype, consisting of transient HI during the neonatal period, followed by progressive beta-cell dysfunction, and subsequent DM in adulthood [4]. However, the precise mechanisms underlying this age-dependent clinical presentation remain unclear. Previous studies have suggested that persistent HI resulting from the adenosine triphosphate-sensitive potassium (K-ATP) channel abnormalities leads to beta-cell apoptosis [11]. This observation suggests that persistent HI may eventually trigger beta-cell apoptosis and subsequent diabetes. A similar pathological mechanism might underlie the transition from HI to diabetes with advancing age in patients with *HNF4A* variants.

On the other hand, there is a phenotypic discrepancy regarding the predictability of disease severity. The clinical features of our patient, specifically the birth weight of +1.5 standard deviation and the responsiveness to diazoxide, are highly representative of the *HNF4A* mutation spectrum. According to a recent multicenter study, *HNF4A* variant carriers frequently present with macrosomia and require medical intervention for neonatal HI [12]. Our patient’s data align with their reported cohort averages, further validating the use of birth weight as a clinical "red flag" for early molecular screening. However, if birth weight reflects the severity of prenatal HI, then the clinical progression to diabetes should, in theory, be predictable by neonatal parameters such as birth weight, GIR, and the required dose of diazoxide. Moreover, an earlier diagnosis in a parent suggests a more rapid progression to insulin deficiency. Interestingly, as summarized in Table 3, which compares our case with previously reported HNF4A cohorts, no clear correlation is observed between these neonatal parameters and the age of parental diabetes diagnosis. This lack of correlation is consistent with our case, where the infant’s relatively mild HI contrasts with the mother’s relatively early onset of diabetes (17 years). Therefore, while birth weight is a reliable indicator of neonatal HI, it may not necessarily predict the long-term diabetic clinical course. The influence of genetic modifiers other than HNF4A, as well as environmental factors, must be considered. Further studies are required to fully elucidate the molecular basis of this uncommon biphasic clinical course.

**Table 3:** Clinical and genetic comparison of the present case with previously reported HNF4A variant cases. This comparative table highlights the clinical spectrum of the present case alongside previously reported cases of *HNF4A* variants. Key clinical features, such as increased BW relative to GA and the requirement for DZX to manage neonatal HI, are consistent across the cohorts. The inheritance patterns and the onset of diabetes in parents are also compared, emphasizing the varied genetic landscape of *HNF4A* variants, with our case specifically involving a novel variant in exon 6. HNF4A, hepatocyte nuclear factor 4 alpha; BW, birth weight; GA, gestational age; GIR, glucose infusion rate; HI, hyperinsulinism; DZX, diazoxide; DM, diabetes mellitus; Pat, paternal; Mat, maternal.

Parameter	Kapoor et al. [10]			Arya et al. [13]	Chandran et al. [9]	Present case
BW (g)	5,900	4,200	4,055	3,610	3,592	3,520
GA (weeks)	39	37	36	37	37	39
Max GIR (mg/kg/min)	25	12.5	11	17.5	16	8.7
Max DZX dose (mg/kg)	10	10	6	5	10	5
Onset of DM in the parent (years)	26	23	31	17	15	17
Inheritance mode	Pat	Pat	Mat	Mat	Pat	Mat
Variant	L330fsdel17ins9	c.264-21A>G	Y16X	S419X	D345Y	A222P
Variant location	Exon 9	Exon 3	Exon 1D	Exon 10	Exon 8	Exon 6

*HNF4A* variants do not appear to impair the function of the K-ATP channel in pancreatic beta cells, which may explain the characteristic drug sensitivity observed in affected individuals. Neonatal HI associated with *HNF4A* variant is often responsive to diazoxide, a K-ATP channel opener [11], whereas DM in adulthood frequently responds well to sulfonylurea therapy [14]. In the present case, the mother of the patient had been receiving intensive insulin therapy prior to the genetic diagnosis of MODY1. Identification of the *HNF4A* variant suggests the potential for reducing insulin therapy and considering alternative treatment options. Given concern regarding the risk of hypoglycemia associated with sulfonylurea, incretin-based therapies, such as DPP-4 inhibitors and glucagon-like peptide-1 receptor agonists, which are associated with a lower risk of hypoglycemia, have been explored as alternatives therapeutic approaches [15]. In this case, a DPP-4 inhibitor was initiated following the diagnosis of MODY1, and the patient’s subsequent glycemic course will be carefully monitored.

Identification of the *HNF4A* variant in this family provided significant clinical benefits by expanding therapeutic options for the mother. Generally, genetic testing for autosomal dominant disorders requires careful consideration because of potential issues within the family and the risks associated with diagnosing asymptomatic carriers. However, in the present case, genetic analysis was feasible because the mother, who was suspected to harbor the *HNF4A* variant, had already developed DM. This provided a genetic significance for diagnosis, even for the mother. Furthermore, the mother expressed interest in pursuing genetic testing to clarify her variant status and re-evaluate her therapeutic strategy, and she subsequently provided informed consent. These findings suggest that genetic analysis may be considered in suspected *HNF4A*-associated disorders when the individual has already developed the disease and when the results are likely to influence clinical management.

The primary limitation of this report is that the pathogenicity of the novel variant was estimated solely through in silico analysis. While in vitro or in vivo functional studies would provide more robust evidence, such evaluations were not feasible due to technical constraints. We anticipate that future molecular biological investigations will further elucidate the functional impact and pathogenicity of this variant.

## Conclusions

The diagnosis of maturity-onset diabetes of the young type 1 (MODY1) in the mother, triggered by hyperinsulinism in her child, provided a significant clinical benefit by allowing for the broadening of therapeutic strategies for her diabetes. Genetic analysis should be actively considered for *HNF4A*-associated disorders when the affected carrier has already developed diabetes, as the results can dramatically expand and optimize therapeutic options.
